# Fermion-induced quantum critical points

**DOI:** 10.1038/s41467-017-00167-6

**Published:** 2017-08-22

**Authors:** Zi-Xiang Li, Yi-Fan Jiang, Shao-Kai Jian, Hong Yao

**Affiliations:** 1 Institute for Advanced Study, Tsinghua University, Beijing, 100084 China; 2 State Key Laboratory of Low Dimensional Quantum Physics, Tsinghua University, Beijing, 100084 China; 3 Collaborative Innovation Center of Quantum Matter, Beijing, 100084 China

## Abstract

A unified theory of quantum critical points beyond the conventional Landau–Ginzburg–Wilson paradigm remains unknown. According to Landau cubic criterion, phase transitions should be first-order when cubic terms of order parameters are allowed by symmetry in the Landau–Ginzburg free energy. Here, from renormalization group analysis, we show that second-order quantum phase transitions can occur at such putatively first-order transitions in interacting two-dimensional Dirac semimetals. As such type of Landau-forbidden quantum critical points are induced by gapless fermions, we call them fermion-induced quantum critical points. We further introduce a microscopic model of SU(*N*) fermions on the honeycomb lattice featuring a transition between Dirac semimetals and Kekule valence bond solids. Remarkably, our large-scale sign-problem-free Majorana quantum Monte Carlo simulations show convincing evidences of a fermion-induced quantum critical points for *N* = 2, 3, 4, 5 and 6, consistent with the renormalization group analysis. We finally discuss possible experimental realizations of the fermion-induced quantum critical points in graphene and graphene-like materials.

## Abstract (web summary)

Quantum phase transitions are governed by Landau-Ginzburg theory and the exceptions are rare. Here, Li et al. propose a type of Landau-forbidden quantum critical points induced by gapless fermions in two-dimensional Dirac semimetals.

## Introduction

Fathoming the behavior of quantum matters near quantum phase transitions in strongly correlated many-body systems is among the central and challenging issues in modern condensed matter physics^1^. Owing to Landau and Ginzburg^2^, a prevalent understanding of phase transitions is provided by order parameters whose non-zero expectation value can characterize phases with lower symmetries. Sufficiently close to the transition point, order parameter fluctuations at large distances and long times dominate the physics near such phase transitions and are described by a continuum field theory of order parameters. Combined with Wilson’s renormalization group (RG) theory^3^, this sophisticated Landau–Ginzburg–Wilson (LGW) paradigm for phase transitions has made huge successes in understanding second-order phase transitions in correlated many-body systems including superconductors, density-wave compounds and electronic liquid crystals^4–6^.

Quantum critical points beyond the LGW paradigm have attracted increasing attentions. It is particularly intriguing to identify and understand quantum critical points, which are forbidden according to the Landau criterion—the so-called Landau-forbidden transitions. Remarkably, the theory of deconfined quantum critical points (DQCP)^7^ provides an exotic scenario of realizing a continuous quantum phase transition between two symmetry-incompatible phases, which is putatively first order according to the Landau symmetry criterion. Fractional excitations have an important role in such DQCP^7–11^.

The Landau cubic criterion states that continuous phase transitions are also forbidden when cubic terms of order parameters are allowed by symmetry in the Landau–Ginzburg (LG) free energy. For instance, the quantum three-state Potts model in 2+1 or 3+1 dimensions has been convincingly shown to feature a first-order quantum phase transition^12^, as cubic terms of the *Z*_3_-order parameters are allowed and relevant in the low-energy LG free energy. One may naturally ask the following question: is there any continuous transition that can violate this Landau criterion concerning cubic terms in LG free energy?

**Fig. 1:**
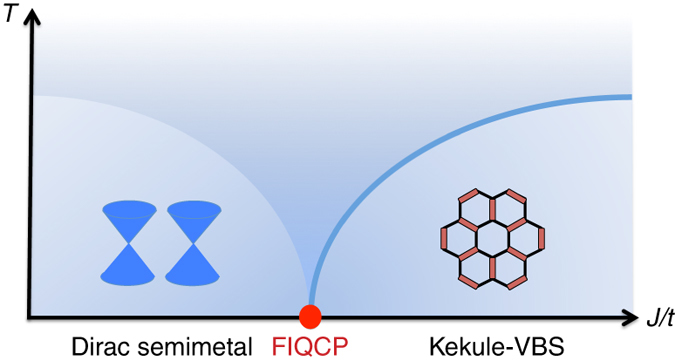
The fermion-induced quantum critical point (FIQCP). According to the Landau cubic criterion, the transition would be putatively first-order because cubic terms of order parameters are allowed by symmetry in the Landau–Ginzburg theory. However, it can be induced to be second order by coupling to massless Dirac fermions. This FIQCP provides a new and generic scenario for transitions violating the Landau cubic criterion

Here we discover an intriguing scenario violating the Landau cubic criterion; namely fermion-induced quantum critical points (FIQCP) are second-order quantum phase transitions induced by coupling gapless fermions to fluctuations of order parameters whose cubic terms appear in the Landau–Ginzburg theory. To be more explicit, we consider a quantum phase transition between Dirac semimetals in two dimensions^13–18^ and Kekule valence bond solids (Kekule-VBS)^19–22^ with *Z*_3_ symmetry-breaking, where cubic terms are allowed in the LG free energy, as schematically shown in Fig. 1. We perform RG analysis to show that this putative first-order phase transition can be driven to a continuous phase transition by fluctuations of gapless Dirac fermions. Our RG calculations are controlled by large-*N* expansions where *N* is the number of flavors of four-component Dirac fermions. Remarkably, the RG results identify a stable fixed point with vanishing cubic terms, which corresponds to a continuous phase transition between Dirac semimetals and Kekule-VBS, namely an FIQCP. To confirm the FIQCP obtained in the RG analysis, we consider microscopic models of *SU*(*N*) fermions on the honeycomb lattice featuring the designed quantum phase transitions. No matter *N* is even or odd, quantum Monte Carlo^23–26^ simulations of these models can be made sign-problem-free by employing the Majorana method recently proposed by us in ref. ^27^. By large-scale sign-problem-free Majorana quantum Monte Carlo (MQMC) simulations, we show convincing evidences that the quantum phase transition between the Dirac semimetals and Kekule-VBS is continuous for *N* = 2, 3, 4, 5 and 6. The emergence of rotational symmetry at the transition reveals that this phase transition falls in chiral XY universality^28, 29^. We obtain various critical exponents at the FIQCP in MQMC simulations. Remarkably, the critical exponents derived from RG analysis reasonably agree with the ones obtained from our MQMC simulations, which strongly suggests that the FIQCPs are robust.

## Results

### RG analysis

We begin by constructing the low-energy field theory describing the quantum phase transition. At low-energy and long-distance near the transition, the system can be described by Dirac fermions, fluctuating order parameters, and their couplings: *S* = *S*_*ψ*_ + *S*_*ϕ*_ + *S*_*ψϕ*_. The action for Dirac fermions (on honeycomb lattice) is given by:

1 $${S_\psi } = {\int} {{{\rm d}^3}x {\psi ^\dag }\left[ {{\partial _\tau } - v\left( {i{\sigma ^x}{\tau ^z}{\partial _x} + i{\sigma ^y}{\tau ^0}{\partial _y}} \right)} \right]\psi } ,$$

where *τ* ^*i*^ (*σ* ^*i*^) Pauli matrices operate in valley (sublattice) space, *v* denotes the Fermi velocity, and ${\psi ^\dag }(x) = \left( {\psi _{{\bf{K}}A}^\dag (x),\psi _{{\bf{K}}B}^\dag (x),\psi _{ - {\bf{K}}A}^\dag (x),\psi _{ - {\bf{K}}B}^\dag (x)} \right)$ is the four component fermion creation operator with $\pm {\bf{K}} = \pm \left( {\frac{{4\pi }}{3},0} \right)$ denoting valley momenta of Dirac points and *A*,*B* labeling sublattices. Note that the spin index *ν* = 1, …, *N* is implicit in the action above; for spin-1/2 electrons in graphene *N* = 2.

The Kekule-VBS order breaks lattice translational symmetry with wave vectors ±2**K** and also the *C*_3_ rotational symmetry (see Supplementary Note 1 for details). The most general but symmetry constrained action describing the order-parameter fluctuations up to the fourth order is given by

2 $${S_\phi } = {\int} {{{\rm d}^{3}}x\left[ {{{\left| {{\partial _\tau }\phi } \right|}^2} + {c^2}{{\left| {\nabla \phi } \right|}^2} + r{{\left| \phi \right|}^2} + b\left( {{\phi ^3} + {\phi ^{ * 3}}} \right) + u{{\left| \phi \right|}^4}} \right],}$$

where *ϕ*(*x*) ≡ *ϕ*_2**K**_(*x*) is a complex order parameter and *c*, *r*, *b* and *u* are real constants. According to the Landau criterion, the cubic terms above should render a first-order transition. Indeed, the action in Eq. (2) describes an effective field theory of quantum three-state Potts model, which supports a weakly first-order quantum phase transition in 2 + 1 dimensions^12, 30, 31^. However, as we shall show below, the coupling between the gapless Dirac fermions and order-parameter fluctuations will dramatically change this scenario by rendering the putative first-order transition into a continuous one. The Kekule-VBS ordering can gap out the Dirac fermions and the coupling between them reads

3 $${S_{\psi \phi }} = g{\int} {{{\rm d}^{3}}x\left( {\phi {\psi ^\dag }{\sigma ^x}{\tau ^ + }\psi + h.c.} \right)} ,$$

where *τ* ^+^ = (*τ* ^*x*^ + *iτ* ^*y*^)/2 and *g* labels the Yukawa-like coupling strength. It is noteworthy that, although the fifth-order term *u*_5_(*ϕ* ^3^ + h.c.)|*ϕ*|^2^, which is relevant at Gaussian fixed point in 2 + 1 dimensions, and the sixth-order term ${u_6}{\left| \phi \right|^6} + u_6\prime \left( {{\phi ^6} + {\rm h.c.}} \right)$, which are marginal, are allowed in the action by symmetry, these terms are irrelevant and can be safely omitted for *N* > 1/2 at the FIQCP fixed point, as we shown explicitly in Supplementary Note 1.

To answer whether the FIQCP occurs in the quantum phase transition, we perform RG analysis of the the effective field theory describing the phase transition. For simplicity, we employ dimensionless coupling constants $\left ( {\tilde r}, \, {{\tilde g}^2},\,{{\tilde b}^2}, \, {\tilde u} \right)$ (see Supplementary Note 1 for details). Integrating out the fast modes in the momentum-shell Λ*e* ^−*l*^ < *p* < Λ yields a set of RG equations, where *l* > 0 parameterizes momentum-shell and Λ is the ultraviolet cutoff. We implement a large-*N* expansion in calculations where *N* is number of fermion species (*N* = 2 for spin-1/2 electrons in graphene). As long as ${\tilde g^2}$ stays non-zero at the infrared, the RG is controlled by a small parameter 1/*N*. A second-order critical point should have only one relevant direction $\tilde r$, whereas those fixed points having more than one relevant directions are multi-critical points or indicate first-order transitions. In other words, a second-order critical point is stable under all perturbations in critical surface $\tilde r \equiv {\tilde r_c}\left( {{{\tilde g}^2},{{\tilde b}^2},\tilde u} \right)$, and, in particular, the cubic terms should be irrelevant.

**Fig. 2:**
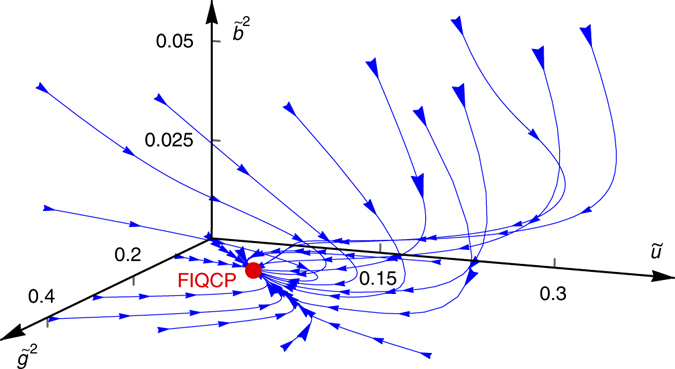
The renormalization group flow of coupling constants. From The only stable fixed point in the critical surface *r* = *r*_c_ is denoted by the red point, which is the Gross–Neveu–Yukawa fixed point representing an FIQCP. Moreover, Lorentz symmetry emerges at the FIQCP as velocities of fermions and bosons flow to the same value

By solving the RG equations, we find only one stable fixed point with ${{{\tilde g}^{ * 2}} { >0}}$ on the critical surface, $\left( {{{\tilde g}^{ * 2}},{{\tilde b}^{ * 2}},{{\tilde u}^ * }} \right) \approx \left( {\frac{2}{{\pi N}},0,\frac{2}{{\pi N}}} \right)$, for *N* > 1/2 (see Supplementary Note 1 for details). Moreover, the Fermi velocity and the boson velocity flow to the same value at the fixed point, indicating a Gross–Neveu–Yukawa (GNY) fixed point with emergent Lorentz symmetry. The flow of coupling constants near the stable GNY fixed point for *N* = 3 is shown in Fig. 2, where the red point denotes the GNY fixed point. In the vicinity of the GNY fixed point^15^, the linearized RG equations are given by (we set *v* = *c* = 1 for simplicity)

4 $$\frac{{{\rm d}\delta {{\tilde g}^2}}}{{{\rm d}l}} = - \delta {\tilde g^2} - \frac{9}{{2N}}\delta {\tilde b^2},$$

5 $$\frac{{{\rm d}\delta {{\tilde b}^2}}}{{{\rm d}l}} = - \frac{9}{N}\delta {\tilde b^2},$$

6 $$\frac{{{\rm d}\delta \tilde u}}{{{\rm d}l}} = \frac{{18}}{N}\delta {\tilde g^2} + \frac{{99}}{N}\delta {\tilde b^2} - \left( {1 + \frac{{18}}{N}} \right)\delta \tilde u,$$

from which it is obvious that the fixed point is a stable one as perturbations around the GNY point are irrelevant. The critical exponents at the GNY fixed point are given by $\eta = \frac{N}{{N + 1}},{\nu ^{ - 1}} = 2 - \frac{{1 + 4N + \sqrt {1 + 38N + {N^2}} }}{{5\left( {1 + N} \right)}}$. At the GNY fixed point, *ϕ* ^5^ (also *ϕ* ^6^) is irrelevant and can be safely neglected near the quantum phase transition for analyzing the FIQCP, as shown in the Supplementary Note 1.

The existence of the stable GNY fixed point in the large-*N* RG analysis implies that the quantum phase transition is a continuous one with vanishing cubic terms and emergent rotational symmetry, namely an FIQCP for relatively large *N*. It is worth mentioning that, if *N* = 0, the theory becomes a purely bosonic system, which features a first-order phase transition as shown many years ago^12^, consistent with the Landau cubic criterion. Moreover, for *N* = 1/2, if the transition were be a continuous one, the critical point would feature an emergent spacetime supersymmetry (SUSY)^32–34^. Because of the emergent SUSY, the scaling dimension of the cubic term *ϕ* ^3^ is known exactly to be 2, which is less than the spacetime dimension, implying that the cubic term is relevant at the SUSY fixed point, and that it is in contradiction with the assumption that a continuous transition occurs for *N* = 1/2. Consequently, the transition for *N* = 1/2 must be first-order, which is an exact result! Our RG analysis predicts a critical *N*_c_ (*N*_c_ > 1/2) such that for *N* > *N*_c_ the gapless fermions are able to drive such a putative first-order transition into a continuous one, and that for *N* < *N*_c_ the putative first-order transition survives. As the RG analysis is controlled by the 1/*N* expansion, determining the exact value of such critical *N*_c_ is beyond the RG scheme here. Sign-problem-free MQMC simulations below give an upper bound of *N*_c_, namely *N*_c_ < 2, as the simulations convincingly show that the FIQCP occurs for *N* = 2, 3, 4, 5 and 6.

### MQMC simulations

In order to confirm the scenario of FIQCP obtained in the RG analysis above, we introduce a sign-problem-free model of SU(*N*) fermions^35–42^ on the honeycomb lattice, which features a quantum phase transition between Dirac semimetals and the Kekule-VBS phase, as follows:

7 $$H = - t\mathop {\sum}\limits_{\left\langle {ij} \right\rangle } {\left[ {c_{i\alpha }^\dag {c_{j\alpha }} + {\rm h.c.}} \right] - \frac{J}{{2N}}\mathop {\sum}\limits_{\left\langle {ij} \right\rangle } {{{\left[ {c_{i\alpha }^\dag {c_{j\alpha }} + {\rm h.c.}} \right]}^2}} } ,$$

where the summation over spin species *α* = 1, …, *N* is implicitly assumed, $c_{i\alpha }^\dag$ is the creation operator of fermions with spin index *α* on site *i*, *t* is the hopping amplitude and *J* is the strength of interactions. Hereafter, we se*t t* = 1 as the energy unit. The low-energy physics of non-interacting *SU*(*N*) fermions at half-filling in Eq. (7) can be described by *N* massless four-component Dirac fermions. The system can undergo a quantum phase transition from Dirac semimetals to the Kekule-VBS phase as the interaction *J* is increased. Most remarkably, no matter *N* is odd or even, this model is free from infamous fermion-sign-problem^43–49^ when the Majorana representation^27^ is used, which allows us to do unbiased simulations to investigate the nature of this quantum phase transition in systems with large lattice sizes.

As we are interested in quantum phase transitions, we use projector QMC^50, 51^ to explore ground-state properties of the model in Eq. (7). To study the transition into the Kekule-VBS phase, we calculate the structure factor of VBS order parameters by MQMC: ${S_{{\rm{VBS}}}}({\bf{k}},L) = \frac{1}{{{L^4}}}\mathop {\sum}\nolimits_{i,j} {{{\rm e}^{i{\bf{k}} \cdot \left ( {{{\bf{r}}_i} - {{\bf{r}}_j}} \right )}} \left \langle {\left ( {c_i^\dag {c_{i + \delta }} + {\rm h.c.}} \right ) \left ( {c_j^\dag {c_{j + \delta }} + {\rm h.c.}} \right )} \right \rangle }$, where the system has 2 × *L* × *L* sites with periodic boundary condition and *δ* labels the direction of a nearest-neighbor bond; the Kekule-VBS order parameter Δ_VBS_ can be obtained through $\Delta _{{\rm{VBS}}}^{2} = {\rm{lim}}_{L \to \infty } \,{S_{{\rm{VBS}}}}({\bf{K}},L)$, where **K** is the VBS ordering vector. It is a finite value when the system lies in the Kekule-VBS phase.

**Fig. 3:**
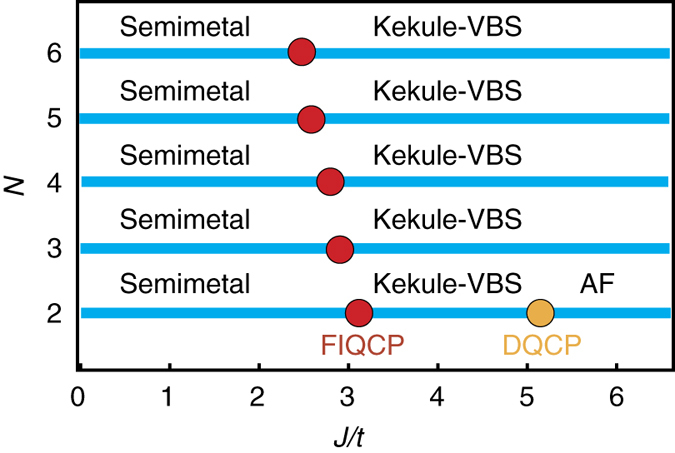
The quantum phase diagram obtained from QMC simulations. The quantum phase diagram of the models of SU(*N*) fermions on the honeycomb lattice, for *N* = 2, 3, 4, 5 and 6, is obtained by sign-problem-free Majorana quantum Monte Carlo (QMC) simulations. For *N* = 2, 3, 4, 5 and 6, the system encounters a continuous transition violating the Landau cubic criterion, dubbed as fermion-induced quantum critical point (FIQCP) here, between the Dirac semimetals to the Kekule valance bond solid (Kekule-VBS) phase. For *N* = 2, the quantum phase transition between the Kekule-VBS and the antiferromagnetic (AF) phases is a deconfined quantum critical point (DQCP)

**Fig. 4:**
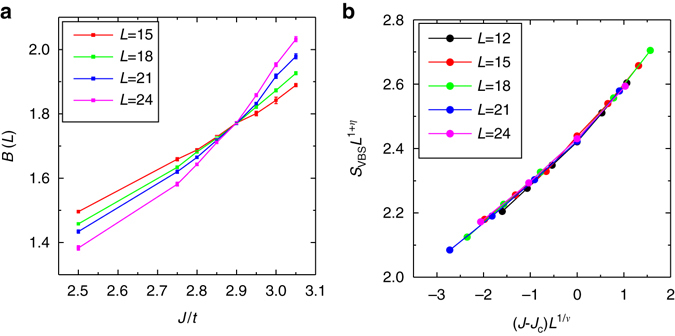
The Binder ratio and data collapse analysis. **a** The Binder ratio with different *J*/*t* and different *L* up to *L* = 24 for *N* = 3 is obtained from sign-problem-free Majorana QMC; the Kekule-VBS phase transition occurs at *J*_c_/*t* ≈ 2.9. **b** Data collapsing according to the scaling relation is used to determine *ν*. From the fitting, *ν* = 1.07 for *N* = 3

We perform large-scale MQMC simulations for *N* = 2, 3, 4, 5 and 6 with *L* = 12, 15, 18, 21 and 24. For simplicity, we shall show *N* = 3 results here and the details about other *N* can be seen in Supplementary Note 2. As shown in Fig. 4a, the critical value *J*_c_ can be obtained through the Binder ratio^26^ of Kekule-VBS order: $B(L) \equiv \frac{{{S_{{\rm{VBS}}}}({\bf{K}},L)}}{{{S_{{\rm{VBS}}}}({\bf{K}} + \delta {\bf{k}},L)}}$, where $\left| {\delta {\bf{k}}} \right| = \frac{{2\pi }}{L}$. At the putative critical point, the Binder ratios of different *L* should cross at the same point for sufficiently large *L*. From MQMC simulations, we calculated the Binder ratio around the Kekule-VBS phase transitions and obtained *J*_c_ for *N* = 2, 3, 4, 5 and 6, as shown in Fig. 3. The case of *N* = 3 is shown in Fig. 4a where *J*_c_ ≈ 2.9*t*, whereas the results for other *N* are shown in Supplementary Fig. 1.

To better answer the question whether the Kekule-VBS transition is first-order or continuous, we further investigate the critical behaviour around the transition. Two independent critical exponents, *η* and *ν*, can be obtained by MQMC simulations and other critical exponents such as *β* may be obtained from *η* and *ν* through hype-scaling relations^52^. The critical exponents *η* and *ν* satisfy the following scaling relation: ${S_{{\rm{VBS}}}}({\bf{K}},L) = {L^{ - z - \eta }}{\cal F}\left( {{L^{1/\nu }}\left( {J - {J_{\rm{c}}}} \right)} \right)$ for *J* close to *J*_c_ and relatively large *L*; we assume the dynamical exponent *z* = 1 mainly because of massless Dirac fermions. First, we obtain *η* by plotting *S*_VBS_(**K**, *L*) at *J* = *J*_c_ vs. *L* in a log–log way and then fitting it to a linear function with slope −(1 + *η*), as shown in Supplementary Fig. 2. Second, there exists an appropriate value of *ν* such that different points (*S*_VBS_(**K**, *L*)*L* ^1+*η*^, *L* ^1/*ν*^(*J*−*J*_c_)) of different *J* around *J*_c_ and different *L* should collapse on a single unknown curve ${\cal {F}}$, as shown in Fig. 4b. For *N* = 3, such finite-size scaling analysis gives rise to *η* ≈ 0.78 and *ν* ≈ 1.07 as shown in Fig. 4. The finite-size scaling analysis for other *N* is shown in Supplementary Fig. 3.

We summarize the results of *η* and *ν* for *N* = 2, 3, 4, 5 and 6 obtained from QMC simulations and RG analysis, respectively, in Table 1. One can see that the values of *η* obtained by QMC and RG are in very good agreement with each other. The agreement in *ν* is not as good as *η*, but should become better for larger *N* as the RG analysis is performed in large-*N* expansion. The reasonable agreement between RG and QMC results for *N* = 2, 3, 4, 5 and 6 convincingly suggests that the quantum phase transition between the Dirac semimetals and the Kekule-VBS phases for *N* ≥ 2 is an FIQCP. The irrelevance of the cubic terms of the VBS order parameter at the FIQCP is further evidenced by the emergence of order-parameter U(1) symmetry at the quantum phase transition, as shown in the Supplementary Fig. 4.

**Table 1:** The critical exponents

*N*	Method	*η*	*ν*
2	Large-*N* (present)	0.67	1.25
	QMC (present)	0.71(3)	1.06(5)
	4 − $\epsilon$, Two-loop^28^	0.67	0.94
3	Large-*N* (present)	0.75	1.26
	QMC (present)	0.78(2)	1.07(4)
	4 − $\epsilon$, Two-loop^28^	0.77	0.96
4	Large-*N* (present)	0.80	1.25
	QMC (present)	0.80(4)	1.11(3)
	4 − $\epsilon$, Two-loop^28^	0.82	0.97
5	Large-*N* (present)	0.83	1.23
	QMC (present)	0.85(4)	1.07(2)
	4 − $\epsilon$, Two-loop^28^	0.86	0.97
6	Large-*N* (present)	0.86	1.22
	QMC (present)	0.87(4)	1.06(3)
	4 − $\epsilon$, Two-loop^28^	0.88	0.98

*η* and *ν* at the FIQCPs obtained in the present work by QMC and RG (large-*N*), respectively, for *N* = 2, 3, 4, 5 and 6. The comparisons with ref. ^28^ are also shown

## Discussion

One possible material candidate to realize FIQCP is graphene. Because of its spin-1/2 degree of freedom, graphene hosts *N* = 2 Dirac fermions. It would be intriguing to observe FIQCPs in graphene-like systems. Indeed, it was reported that Kekule-VBS ordering has been experimentally observed under certain conditions in graphene^53^. According to our RG analysis and MQMC simulations, we predict that the quantum phase transitions into Kekule-VBS in the *N* = 2 Dirac systems (i.e. graphene-like materials) are FIQCPs. Other materials that might feature an FIQCP include 2H-TaSe_2_, for which the 3 × 3 charge-density wave ordering seems to be a continuous transition^54^ even though according to Landau it would be first order.

We have shown that a putative first-order phase transition can be driven to a continuous one by coupling to *N* massless Dirac fermions in 2 + 1 dimensions, from both sign-problem-free Majorana QMC simulations with *N* ≥ 2 and the large-*N* RG analysis. To the best of our knowledge, it is for the first time that an FIQCP in 2 + 1 dimensions is convincingly established as a quantum phase transition violating the Landau cubic criterion. Our proposal of FIQCP in the present study was further confirmed by subsequent works, which find interesting examples of FIQCP in other types of theories^55, 56^. We would also like to mention that our simulations show evidences of a DQCP between the Kekule-VBS and antiferromagnetic phases occurring in the *N* = 2 model in Eq. (7), as shown in Fig. 3. We believe that our study has provided new insights toward a unified understanding of quantum critical points and shall pave a new avenue to understand exotic quantum phase transitions beyond the conventional LGW paradigm^57^.

## Methods

### RG analysis

The RG equations are obtained by one-loop large-*N* calculations in 2 + 1 dimensions.

### Quantum Monte Carlo

The projector QMC calculation is carried out to explore the ground-state properties of model Eq. (7). In projector QMC, the ground state is obtained through projection: $\left| {{\psi _0}} \right \rangle = {\rm lim} _{\Theta \to \infty } {{\rm e}^{ - \Theta H}}\left| {{\psi _T}} \right \rangle$, where $\left| {{\psi _T}} \right \rangle$ is a trial wave function and is chosen to be the ground state of the non-interacting part of in Eq. (7). Because of the absence of sign-problem in Majorana representation, we can perform large-scale QMC simulations with large system sizes and sufficiently large Θ. In our simulations, we use Θ = 40 and also have checked that results stay nearly the same for larger Θ. We set discrete imaginary-time step Δ*τ* = 0.04 and verified that the results do not change within our statistics errors if we use smaller Δ*τ*. Simulations are performed in honeycomb lattice of linear sizes *L* = 12, 15, 18, 21 and 24 with *N* = 2*L* ^2^ sites. Binder ratio and data collapse techniques are implemented to identify the accurate QCP and extract critical exponents.

### Data availability

The data that support the findings of this study are available from the corresponding author (H.Y.) upon request.

## Electronic supplementary material

Supplementary Information
