# Supplementary material for: Fermion-induced quantum critical points
Source: Nat Commun. 2017 Aug 22;8:314. doi: 10.1038/s41467-017-00167-6 (PMC5566446; doi:10.1038/s41467-017-00167-6)
Supplement: Supplementary file 1 — Supplementary Information [file 41467_2017_167_MOESM1_ESM.pdf]

File name: Supplementary Information
Description: Supplementary Figures and Supplementary Notes

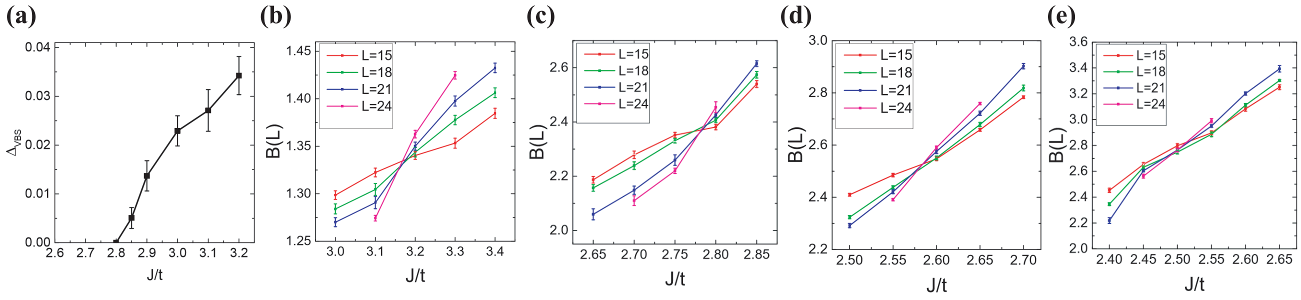

Supplementary Figure 1. | **The Kekule-VBS order parameter and Binder ratio.** (**a**) The Kekule valance bond solid (Kekule-VBS) order parameter versus J/t for $N = 4$. The Kekele-VBS order parameter is extracted by fitting VBS structure factor using second-polynomials in $1/L$. The standard method of least square is employed to fit the second-polynomials function of $1/L$. The error bar denotes the standard error of the intercept of second-polynomials function. (**b-e**) The Binder ratio results for different N: (**b**) $N = 2$, (**c**) N=4, (**d**) N=5, (**e**) $N = 6$; The phase transition point of J/t is the crossed point of Binder ratio curve with different L.

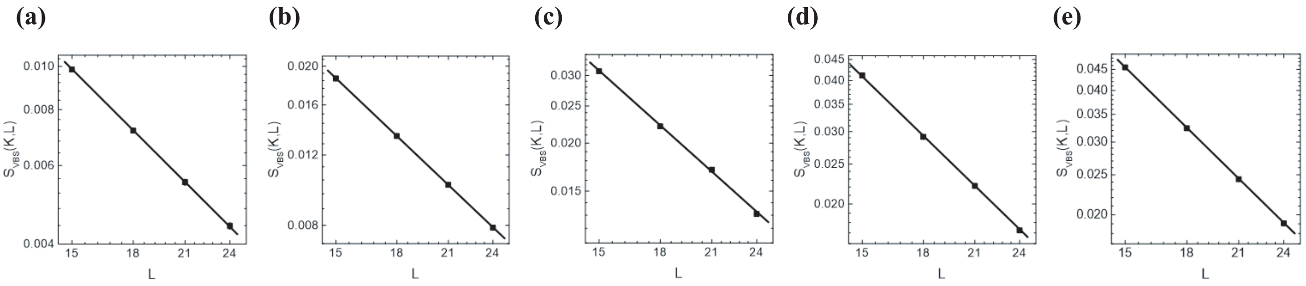

Supplementary Figure 2. | **The critical exponent** $\boldsymbol{\eta}$**.** The critical exponent η can be obtained from the fitting in the log-log plot of the Kekule-VBS structure factor versus L at $J = J_c$ for different N. (**a**) $N = 2$. (**b**) $N = 3$. (**c**) $N = 4$. (**d**) $N = 5$. (**e**) $N = 6$.

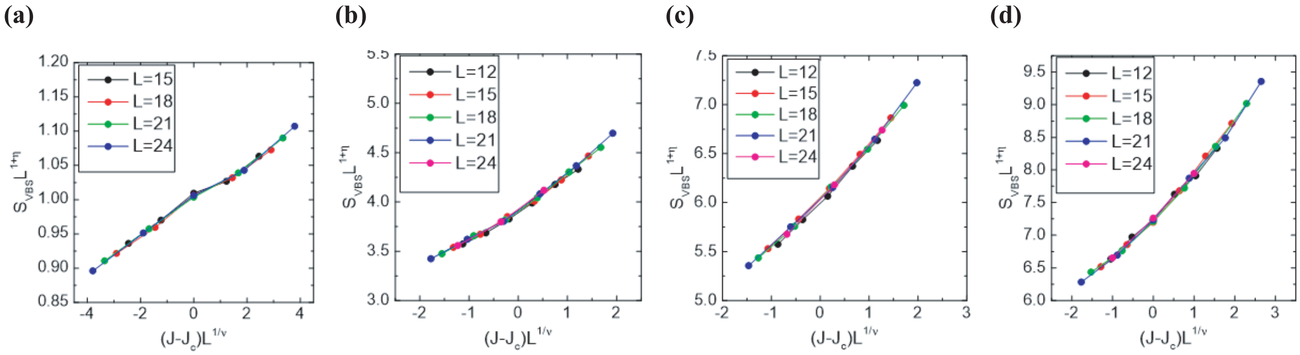

Supplementary Figure 3. | **Data collapse results for different *N*.** The critical exponent ν can be obtained through data collapse. (**a**) $N = 2$. (**b**) $N = 4$. (**c**) $N = 5$. (**d**) $N = 6$.

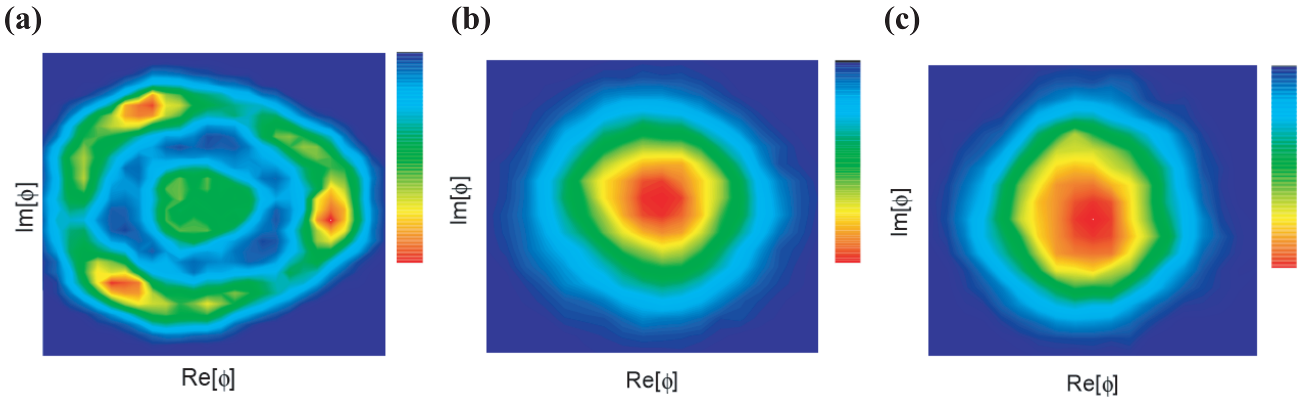

Supplementary Figure 4. | **The color-coded histogram of the VBS order parameter.** The color-coded histogram of the VBS order parameter in the system with $L = 15$. Red color means the concurrence probability of VBS order parameter $P(\mathrm{Re}(\phi), \mathrm{Im}(\phi))$ is high, while blue color means concurrence probability of VBS order parameter $P(\mathrm{Re}(\phi), \mathrm{Im}(\phi))$ is low. (**a**) $J/t = 4.0$ for $N = 3$: the system is located in the VBS ordered phase. The VBS order parameter exhibits C_3 symmetry. (**b**) $J/t = 2.95$ for $N = 3$: the system is located at transition point from the Dirac semimetal to the Kekule-VBS phase. The VBS order parameter exhibits an emergent $U(1)$ symmetry. (**c**) $J/t = 3.2$ for $N = 2$: the system is located at transition point from semimetal to VBS phase, at which an emergent $U(1)$ symmetry occurs.

Supplementary Note 1: Renormalization group analysis

As mentioned in the main text, at the quantum phase transition point, the low-energy and long-distance physics can be described by the massless Dirac fermions, the fluctuating Kekule valance bond solid (Kekule-VBS) order parameters, and the couplings between fermions and order parameter fields: $S = S_\psi + S_\phi + S_{\psi\phi}$. The action for massless Dirac fermions on the honeycomb lattice is given by:

$$S_\psi = \int \frac{d^d k}{(2\pi)^d} \psi^\dagger[-iw + v(k_x\sigma^x\tau^z + k_y\sigma^y\tau^0)]\psi, \tag{1}$$

where τ^i (σ^i) are Pauli matrices with valley (sublattice) indices, and $\psi^\dagger = (\psi^\dagger_{\mathbf{K}A}, \psi^\dagger_{\mathbf{K}B}, \psi^\dagger_{-\mathbf{K}A}, \psi^\dagger_{-\mathbf{K}B})$ is fermion creation operators around the Dirac points $\pm\mathbf{K} = \pm(\frac{4\pi}{3}, 0)$.

The Kekule-VBS order parameter possesses $2\mathbf{K}$ momentum and serves a two-dimensional irreducible representation of the C_3 group, which is generated by translation operators $T_1, T_2 \equiv T_1^2$, where T_1 denotes translating by lattice vector $\mathbf{e_1} = (1, 0)$ and T_2 denoting $\mathbf{e_2} = (\frac{1}{2}, \frac{\sqrt{3}}{2})$. Static Kekule-VBS ordering can generate a mass for Dirac fermions. Thus, the Kekule-VBS order parameters is given by $\phi_{2\mathbf{K}} = \langle\psi^\dagger_{\mathbf{K}}\sigma^x\psi_{-\mathbf{K}}\rangle$. The transformation laws for the Kekule-VBS order parameters read:

$$T_1(\phi_{2\mathbf{K}}) = e^{i\frac{4\pi}{3}}\phi_{2\mathbf{K}}, \tag{2}$$
$$T_2(\phi_{2\mathbf{K}}) = e^{i\frac{2\pi}{3}}\phi_{2\mathbf{K}}, \tag{3}$$
$$P_x(\phi_{2\mathbf{K}}) = \phi^*_{2\mathbf{K}}, \tag{4}$$
$$P_y(\phi_{2\mathbf{K}}) = \phi_{2\mathbf{K}}, \tag{5}$$

where P_i is the reflection operator sending i to $-i$. The most general action for the Kekule-VBS order parameter allowed by the symmetries up to fourth-order is given by:

$$S_\phi = \int d^d x \Big[|\partial_\tau\phi|^2 + c^2|\nabla\phi|^2 + r|\phi|^2 + b(\phi^3 + \phi^{*3}) + u|\phi|^4\Big], \tag{6}$$

where $\phi \equiv \phi_{2\mathbf{K}}$ for simplicity. The cubic terms in the action are allowed by C_3 symmetry and b is a real constant as required by the reflection symmetries. Dictated by symmetry, the most relevant fermion-boson coupling reads:

$$S_{\psi\phi} = g\int d^d x(\phi_{2\mathbf{K}}\psi^\dagger_{\mathbf{K}}\sigma^x\psi_{-\mathbf{K}} + h.c.), \tag{7}$$
$$= g\int d^d x(\phi\psi^\dagger\sigma^x\tau^+\psi + \phi^*\psi^\dagger\sigma^x\tau^-\psi), \tag{8}$$

where $\tau^\pm \equiv \frac{1}{2}(\tau^x \pm \tau^y)$ and we have used the gauge freedom to fix g to be real. For convenience, we define gamma matrix: $\gamma^0 = \sigma^z, \gamma^1 = -\sigma^y\tau^z, \gamma^2 = \sigma^x, \gamma^3 = \sigma^y\tau^x, \gamma^5 = \sigma^y\tau^y$, which have the properties $\{\gamma^\mu, \gamma^\nu\} = 2\delta^{\mu\nu}$, and $\gamma^\pm = \frac{1}{2}(\gamma^3 \pm i\gamma^5)$. In the following, we use Greek letter to denote $0, 1, 2$ and English letter to denote $\pm$. In this convention, the action is given by $S = S_\psi + S_{\phi,0} + S_{\rm int}$ with

$$S_\psi = \int \frac{d^d k}{(2\pi)^d}\bar\psi(-i\gamma^0 k_0 - iv\gamma^i k_i)\psi, \tag{9}$$
$$S_{\phi,0} = \int \frac{d^d k}{(2\pi)^d}\phi^*(k_0^2 + c^2 k_i^2 + r)\phi, \tag{10}$$
$$S_{\rm int} = \int d^d x\left[b(\phi^3 + \phi^{*3}) + u|\phi|^4 + ig(\phi\bar\psi\gamma^+\psi + \phi^*\bar\psi\gamma^-\psi)\right]. \tag{11}$$

where $\bar\psi = \psi^\dagger\gamma^0$ and $k_\mu = (w, k_x, k_y, k_z)$ and $k^2 = k_\mu k_\nu\delta^{\mu\nu}$. The Feynman propagators are given by

$$S(k) = \frac{i(\gamma^0 k_0 + v\gamma^i k_i)}{k_0^2 + v^2 k_i^2}, \tag{12}$$
$$D(k) = \frac{1}{k_0^2 + c^2 k_i^2 + r}. \tag{13}$$

In the following calculation, the gamma matrices have the properties: $\{\gamma^i,\gamma^j\}=2g^{ij},\{\gamma^i,\gamma^\mu\}=0,\{\gamma^\mu,\gamma^\nu\}=2\delta^{\mu\nu}$, where $g^{ij}=(1-\delta^{ij})/2$. Moreover, the trace properties are listed here for reference:

$$\mathrm{Tr}[\gamma^i\gamma^\mu\gamma^j\gamma^\nu]=-4Ng^{ij}\delta^{\mu\nu}, \tag{14}$$

$$\mathrm{Tr}[\gamma^\mu\gamma^\nu\gamma^\rho\gamma^\sigma]=4N[\delta^{\mu\nu}\delta^{\rho\sigma}-\delta^{\mu\rho}\delta^{\nu\sigma}+\delta^{\mu\sigma}\delta^{\nu\rho}], \tag{15}$$

$$\mathrm{Tr}[\gamma^i\gamma^\mu\gamma^j\gamma^\nu\gamma^k\gamma^\rho]=0, \tag{16}$$

$$\mathrm{Tr}[\gamma^i\gamma^\mu\gamma^j\gamma^\nu\gamma^k\gamma^\rho\gamma^l\gamma^\sigma]=4N[\delta^{\mu\nu}\delta^{\rho\sigma}-\delta^{\mu\rho}\delta^{\nu\sigma}+\delta^{\mu\sigma}\delta^{\nu\rho}][g^{ij}g^{kl}-g^{ik}g^{jl}+g^{il}g^{jk}], \tag{17}$$

where we have promoted the spin $SU(2)$ to $SU(N)$. We implement a large-N expansion in calculation. We will see that as long as g^2 stays non-zero at the infrared, the renormalization group (RG) procedure is controlled by $1/N$. This means the critical $\tilde r=\tilde r_c$ is of order $1/N$; consequently setting $\tilde r_c=0$ in calculations will not affect the result in the lowest order. We use a momentum-shell renormalization scheme. Namely, the fast modes in the spherical momentum-shell $\Lambda e^{-l}<p<\Lambda$ are integrated out, giving renormalization to the slow modes with $p<\Lambda e^{-l}$, where $l>0$ is the flow parameter. To accommodate this renormalization effects, various coupling constants begin to run when energy scale changing. The relevant renormalization comes from $S_{\text{eff}}=S_<-\frac{1}{2}\langle S_c^2\rangle_>+\frac{1}{6}\langle S_c^3\rangle_>-\frac{1}{24}\langle S_c^4\rangle_>$ to one-loop level, where $>,<$ denote the fast and slow modes, respectively, and $\langle\cdots\rangle_>$ means taking expectation value in fast mode configurations. Boson self-energy $\Pi(p)$ and fermion self-energy $\Sigma(p)$ are

$$\begin{aligned}\Pi(p)&=\int\frac{d^dk}{(2\pi)^d}\mathrm{Tr}[(ig\gamma^+)S(p+k)(ig\gamma^-)S(k)]-18b^2\int\frac{d^dk}{(2\pi)^d}D(p+k)D(k),\\&=g^2\frac{N\pi}{2v^3}K_d\Lambda^{d-4}l(p_0^2+v^2p_i^2)+b^2\frac{9\pi}{4c^5}K_d\Lambda^{d-6}l(p_0^2-\frac{1}{3}c^2p_i^2),\end{aligned} \tag{18}$$

and

$$\Sigma(p)=-2\int\frac{d^dk}{(2\pi)^d}(ig\gamma^-)S(p+k)(ig\gamma^+)D(k), \tag{19}$$

$$=g^2\frac{\pi}{c(c+v)^2}K_d\Lambda^{d-4}l[\gamma^0(-ip_0)+\frac{2c+v}{3v}\gamma^i(-ivp_i)], \tag{20}$$

where $K_d=\frac{A_{d-1}}{(2\pi)^d}$, and A_d is the surface area of unit d-sphere. Renormalization contribution to three-boson-vertex is

$$\Gamma_{\phi^3}=\Gamma_{\phi^{*3}}=-6bu\int\frac{d^dk}{(2\pi)^d}D^2(k)-\frac{1}{3}\int\frac{d^dk}{(2\pi)^d}\mathrm{Tr}[S(k)(ig\gamma^-)S(k)(ig\gamma^-)S(k)(ig\gamma^-)], \tag{21}$$

$$=-bu\frac{3\pi}{c^3}K_d\Lambda^{d-4}l, \tag{22}$$

and to four-boson-vertex is

$$\begin{aligned}\Gamma_{|\phi|^4}&=-10u^2\int\frac{d^dk}{(2\pi)^d}D^2(k)+144ub^2\int\frac{d^dk}{(2\pi)^d}D^3(k)-324b^4\int\frac{d^dk}{(2\pi)^d}D^4(k)\\&+\int\frac{d^dk}{(2\pi)^d}\mathrm{Tr}[S(k)(ig\gamma^+)S(k)(ig\gamma^-)S(k)(ig\gamma^+)S(k)(ig\gamma^-)],\\&=-u^2\frac{5\pi}{c^3}K_d\Lambda^{d-4}l+ub^2\frac{54\pi}{c^5}K_d\Lambda^{d-6}l+g^4\frac{N\pi}{2v^3}K_d\Lambda^{d-4}l-b^4\frac{405\pi}{4c^7}K_d\Lambda^{d-8}l.\end{aligned} \tag{23}$$

The contribution from integrating out the fast modes to fermion-boson-vertex vanishes because of properties of $\gamma^\pm$ structure. After rescaling $p\to e^{-l}p$, we bring the renormalization effects into the flow of coupling constants. This results in the following RG equations:

$$\frac{dc}{dl}=-\frac{N\pi(c^2-v^2)}{4c^3v^3}\tilde g^2-\frac{3\pi}{2c^6}\tilde b^2, \tag{24}$$

$$\frac{dv}{dl}=-\frac{2\pi(v-c)}{3cv(c+v)^2}\tilde g^2, \tag{25}$$

$$\frac{d\tilde g^2}{dl}=(4-d)\tilde g^2-\frac{9\pi}{4c^5}\tilde b^2\tilde g^2-\left(\frac{N\pi}{2v^3}+\frac{2\pi}{c(c+v)^2}\right)\tilde g^4, \tag{26}$$

$$\frac{d\tilde b^2}{dl}=(6-d)\tilde b^2-\frac{3N\pi}{2v^3}\tilde g^2\tilde b^2-\frac{6\pi}{c^3}\tilde b^2\tilde u-\frac{27\pi}{4c^5}\tilde b^4, \tag{27}$$

$$\frac{d\tilde u}{dl}=(4-d)\tilde u-\frac{N\pi}{v^3}\tilde g^2\tilde u+\frac{N\pi}{2v^3}\tilde g^4-\frac{5\pi}{c^3}\tilde u^2+\frac{99\pi}{2c^5}\tilde u\tilde b^2-\frac{405\pi}{4c^7}\tilde b^4, \tag{28}$$

where we define dimensionless coupling constants: $\tilde{g}^2 = K_d\Lambda^{d-4}g^2, \tilde{b}^2 = K_d\Lambda^{d-6}b^2, \tilde{u} = K_d\Lambda^{d-4}u$. Solving these coupled RG equations in (2+1)-dimensions ($d = 3$), we find four fixed points totally. On one hand, two of them locates in the ($\tilde{b}^2$,$\tilde{u}$) plane with $\tilde{g}^2 = 0$ and are the familiar Gaussian ($\tilde{b}^{*2} = 0$ and $\tilde{u}^* = 0$) and Wilson-Fisher fixed points ($\tilde{b}^{*2} = 0$ and $\tilde{u}^* = 1/(5\pi)$), respectively. Both fixed points are unstable. Since the fermions decouple with order parameters at these fixed point, they are not controlled by $1/N$ and are not relevant in this work.

On the other hand, there are two fixed points with non-zero fermion-boson coupling. But, only one of them is a physically meaningful fixed point with $u^* > 0$ while the other one with $u^* < 0$ is not physically meaningful as the free energy is not bounded from below. At the physical fixed point, the fermion and the boson velocities flow to the same value, i.e., $c^* = v^*$. Thus, we set $c^* = v^* = 1$ below for simplicity. The coupling constants at this fixed point are given by $(\tilde{g}^{*2}, \tilde{b}^{*2}, \tilde{u}^*) = (\frac{2}{\pi(N+1)}, 0, R)$, where $R = \frac{1-N+\sqrt{N^2+38N+1}}{10\pi(N+1)}$. Remarkably, the rotational and Lorentz symmetries emerge at low-energy and long-distance as $c^* = v^*$ and $\tilde{b}^{*2} = 0$, which is a Gross-Neveu-Yukawa (GNY) fixed point corresponding to chiral XY universality. This GNY fixed point is stable for relatively large N_c as indicated by the flow diagram as well as the linearized RG equations given in main text and controls the behaviors at the FIQCP. The scaling fields near this fixed point read $(-1, -\frac{\sqrt{N^2+38N+1}}{N+1}, \frac{3(N+4-\sqrt{N^2+38N+1})}{5(N+1)})$, from which one can find the critical number reads $N_c = \frac{1}{2}$. For $N > N_c$, the GNY fixed point is stable due to the negativity of all scaling fields.

We believe that the second-order phase transition found in Majorana quantum Monte Carlo (MQMC) simulation is controlled by the GNY fixed point. At this fixed point fermion and boson fields will get non-trivial anomalous dimensions:

$$\eta_\phi = \frac{N\pi}{4}\tilde{g}^{*2} + \frac{9\pi}{8}\tilde{b}^{*2} = \frac{N}{2N+2}, \tag{29}$$

$$\eta_\psi = \frac{\pi}{8}\tilde{g}^{*2} = \frac{1}{4(N+1)}. \tag{30}$$

The critical exponent η is directly related to $\eta \equiv 2\eta_\phi = \frac{N}{N+1}$. To determine other critical exponents, we calculate the $\phi^2(x)$ vertex:

$$\Gamma_{|\phi|^2} = -18b^2\int\frac{d^dk}{(2\pi)^d}\frac{1}{(k^2+r)^2} + 4u\int\frac{d^dk}{(2\pi)^d}\frac{1}{k^2+r}, \tag{31}$$

$$= \left(-\frac{9\pi\tilde{b}^2}{(1+\tilde{r})^{3/2}} + \frac{4\pi\tilde{u}}{\sqrt{1+\tilde{r}}}\right)\Lambda^2 l, \tag{32}$$

where $\tilde{r} = \Lambda^{-2}r$. From this contribution, we have $\eta_r = -\eta + \frac{27\pi}{2}\tilde{b}^{*2} - 2\pi\tilde{u}^* = -\frac{1+4N+\sqrt{1+38N+N^2}}{5(1+N)}$. Thus, the critical exponent at the GNY fixed point is given by $\nu^{-1} = 2 + \eta_r$ which gives rise to the formula in the main text.

We consider the insertion of the term $u_5(\phi^3 + h.c.)|\phi|^2$ into the fixed-point action and ask whether it would affect the GNY fixed point or not. The RG equation of u_5 is given by

$$\frac{du_5}{dl} = \left[\frac{1}{2} - \frac{5N}{2(N+1)}\right]u_5 - 8\pi\tilde{u}u_5, \tag{33}$$

where $\tilde{u}$ is the dimensionless coupling constant of the quartic term defined in the main text and l is the flow parameter. For $N > 1/2$, u_5 is irrelevant at the GNY fixed point. Similar calculations also show that $u_6|\phi|^6$ and and $u_6'(\phi^6 + h.c.)$ terms are irrelevant at the GNY fixed point. Thus, these terms can be safely neglected near the quantum phase transition for analyzing the FIQCP.

Supplementary Note 2: MQMC results of the $SU(N)$ models for $N = 2, 3, 4, 5, 6$

In order to confirm the scenario of FIQCP, we perform sign-problem-free MQMC simulations of the $SU(N)$ fermionic model (Eq. 6) on honeycomb lattice for $N = 2, 3, 4, 5, 6$. The largest linear system sizes in the simulations are $L = 24$. We compute Binder ratio to determine phase transition points from semimetal to kekule-VBS phase. The results of Binder ratio for $N = 2, 4, 5, 6$ are shown in Supplementary Figure 1, from which we obtain the quantum critical values of J for different N(shown in Fig. 2). We can see that the critical points of J decrease monotonously as N is increased. This trend is expected because the quantum fluctuations are stronger for smaller N such that the critical values of J are larger than the mean-field value. The critical J_c in the limit of $N \to \infty$ is equal to the mean-field value.

To study the critical behaviour of these transition points for different N, we use the scaling function $S_{\text{VBS}}(\mathbf{K}, L) = L^{-z-\eta}\mathcal{F}(L^{1/\nu}(J - J_c))$ to obtain the critical exponents η and ν. The results are shown in Supplementary Figure 2

and Supplementary Figure 3. It is obvious that for $N = 2, 4, 5, 6$, the structure factor with different L and J can be collapsed to a smooth scaling function by choosing appropriate values of η and ν, which indicates that these transitions are continuous. The critical exponents η and ν of the quantum phase transitions between the Dirac semimetals and the Kekule-VBS for $N = 2, 3, 4, 5, 6$ fermions are summarized in Table. I of the main text.

In order to verify the prediction of emergent $U(1)$ symmetry at the FIQCP obtained in our RG's analysis, we employ the technique of histogram by studying the concurrence probability of VBS order parameter $P(\mathrm{Re}(\phi), \mathrm{Im}(\phi))$. The VBS order parameter ϕ is given by: $\phi = \sum_i (c_i^\dagger c_{i+\delta} + h.c) e^{i2\mathbf{K}\cdot\mathbf{r}_i}$. Our histogram analysis shows that in the Kekule-VBS ordered phase, the concurrence probability $P(\mathrm{Re}(\phi), \mathrm{Im}(\phi))$ exhibits the expected C_3 symmetry. However, at the phase transition point between the Dirac semimetal and the Kekule-VBS phase, the histogram of the VBS order parameter should exhibit an emergent $U(1)$ symmetry if the transition is a continuous one. The QMC results of the histogram both at the transition points and deep in the VBS ordered phase for $N = 2, 3$ are shown in Supplementary Figure 4, respectively. Indeed, the histogram at the transition point exhibit an emergent $U(1)$ symmetry, which provides strong evidences of ruling out the possibility of a first-order transition.
